# Improving Complementary Food Hygiene Behaviors Using the Risk, Attitude, Norms, Ability, and Self-Regulation Approach in Rural Malawi

**DOI:** 10.4269/ajtmh.19-0528

**Published:** 2020-02-24

**Authors:** Kondwani Chidziwisano, Jurgita Slekiene, Hans-Joachim Mosler, Tracy Morse

**Affiliations:** 1 Department of Environmental Health and WASHTED Centre, Polytechnic, University of Malawi, Blantyre, Malawi;; 2 Department of Civil and Environmental Engineering, University of Strathclyde, Glasgow, United Kingdom;; 3 Eawag, Swiss Federal Institute of Aquatic Science and Technology, Dübendorf, Switzerland

## Abstract

The study evaluated the effectiveness of an intervention to improve complementary food hygiene behaviors among child caregivers in rural Malawi. Formative research and intervention development was grounded in the risk, attitude, norms, ability, and self-regulation (RANAS) model and targeted washing hands and kitchen utensils with soap, safe utensil storage, reheating of leftover food, and feeding of children by caregivers. Longitudinal research was applied at baseline and follow-up surveys among 320 caregivers. Determinants of selected behaviors were found, and interventions were developed based on the behavior change techniques aligned with these determinants in the RANAS model. The intervention was delivered over 9 months through group (cluster) meetings and household visits and included demonstrations, games, rewards, and songs. We randomly assigned villages to the control or intervention group. Follow-up results indicated a significant increase in three targeted behaviors (washing kitchen utensils with soap, safe utensil storage, and handwashing with soap) among intervention recipients. Several psychosocial factors differed significantly between the intervention and control groups. Mediation results showed that the intervention had a significant effect on these three targeted behaviors. For handwashing, feelings, others’ behavior in the household, and remembering; for washing kitchen utensils, others’ behavior in the household and difficulty to get enough soap; for safe utensils storage, others’ behavior in the village and remembering mediated the effect of the intervention on the targeted behaviors. The study demonstrated that targeting food hygiene behaviors with a theory-driven behavior change approach using psychosocial factors can improve the behavior of child caregivers in rural Malawi.

## INTRODUCTION

Globally, diarrheal diseases are the second leading cause of deaths after acute respiratory infections among children younger than 5 years, with approximately 424, 000 deaths annually.^1^ Contaminated water, food, and hands have been associated with diarrhea causation in children.^2–4^ Annually, contaminated food alone contributes to 550 million cases of diarrhea, with 230,000 deaths worldwide.^5^ Furthermore, it is estimated that 125,000 deaths occur annually among children younger than 5 years in low- and middle-income countries (LMICs) resulting from the burden of food-borne diseases.^5^

Complementary food hygiene practices have been linked to diarrhea among children in low-income settings.^6,7^ This has been related to unhygienic food preparation and storage environments such as the method of washing utensils,^8^ use of contaminated utensils,^9^ poor storage of food (temperature and covering) and utensils,^10,11^ presence of animals in food preparation and storage areas,^12^ and lack of handwashing at critical times, for example, before food preparation and child feeding.^13–15^ Post-cooking activities (e.g., usage of utensils, handwashing, and storage of food) were identified as the main critical areas to potentially control food contamination in rural Malawi.^16,17^

Despite the significant burden of food-borne diseases in LMICs, little effort has been made to understand and improve food hygiene practices in rural household settings. Such an understanding is important for the promotion of child health programs (e.g., nutrition programs) because complementary feeding, water, sanitation, and hygiene (WASH) have been associated with high risk of growth failure.^18–21^ Despite this, there has been little emphasis on food hygiene in nutrition or child health programming.^22^ Previous research studies have focused on measuring microbial contamination in food with little attention to the development of context-appropriate food hygiene behavior change interventions.^16,17,23–26^ Those which developed and tested food hygiene behavior change interventions^13,27–29^ focused on increasing the level of knowledge as well as provision of WASH infrastructure and did not address the psychosocial determinants integral to the performance of a behavior.

To bring about a behavior change, psychosocial factors that determine a behavior should be explored to understand why people perform particular health behaviors. Such an assessment provides the basis for the development of subsequent effective behavior change interventions.^30,31^ The risk, attitude, norms, ability, and self-regulation (RANAS) model of the behavior change provides detailed psychosocial block factors from a diverse range of psychological theories.^32^ Risk factors include the level of understanding and awareness of the person’s vulnerability and severity of diseases. It also incorporates health knowledge about disease transmission, prevention options, and personal consequences. Attitudinal factors relate to one’s assessment of the beliefs about costs and benefits of a particular behavior and feelings associated with the behavior. Norm factors present the perception of what behavior is performed in society, describing how family and community members, including leaders, approve or disapprove a particular behavior. Ability factors describe an individual’s capacity to practice a particular behavior, which includes its uptake, maintenance, and recovery from drawbacks. Finally, self-regulation factors describe one’s plan on how to maintain a behavior, and it includes how to address barriers to the implementation of the behavior.

The RANAS model has been applied successfully to determine behavioral factors as well as to promote water treatment, sanitation, and handwashing practices in LMICs.^33–36^ Importantly, we used the RANAS model for the first time to identify and inform an intervention centered on the psychosocial factors influencing complementary food hygiene behaviors in rural Malawi.^37,38^

### The present study.

The first aim of this study was to demonstrate the effectiveness of an evidence-based intervention on complementary food hygiene behaviors, such as handwashing with soap, washing kitchen utensils with soap, keeping kitchen utensils in a safe (elevated) place, reheating of leftover food, and child feeding by the caregivers. The second aim of the study was to reveal the underlying mechanisms of the behavior change using a theory-based approach and mediation analysis method. This provides information on the most effective elements of the behavior change intervention when addressing complementary food hygiene behaviors.

We addressed the following research questions in our study:

1. Did target behaviors change because of the intervention?
2. Which psychosocial factors changed between intervention and the control group, and how did these vary?
3. Which psychosocial factors changed because of the intervention and, therefore, mediated the change in behavior?

## MATERIALS AND METHODS

### Study area and design.

The longitudinal study included two surveys at baseline and follow-up in rural Malawi between February 2017 and December 2018. The evidence-based intervention package was implemented from February until October 2018. The study design comprised two arms: one was an intervention arm, while the other served as a control. The intervention arm received the “hygienic family” behavior change intervention package, whereas no intervention was implemented among the control households. The study was conducted in Chikwawa district, which is located in the southern region of Malawi. With a population of 564,684 (of which 16% are younger than 5 years),^39^ the district is divided into 12 traditional authorities (TAs). This study was conducted in three TAs. Generally, households were made of mud walls (59%), thatch roof (77.1%), and had domesticated animals (61%). Separate kitchens were rare (43%) in the area with the majority of food preparation, including cooking, taking place in the household yard. Similar to other districts in Malawi, fire wood is the main source of energy for cooking in rural Chikwawa (90–95%).^40,41^ According to Cohen,^42,43^ an alpha level of 0.05 and small population effect size for analysis of variance (ANOVA) calculations require a sample size of 393 respondents when comparing two groups. However, our study included 320 respondents (i.e., 240 households in the intervention area and 80 households from the control area) who were available at baseline and follow-up surveys as the study was designed to interview the same respondents at both data collection points. The inclusion criteria for a household to be part of the study required that it should be located in the intervention or control area, had a functioning latrine, and resided within a 500-m radius of a functioning borehole to ensure that there were no significant variations in access to water or sanitation infrastructure. In addition, eligible households had a child aged between 4 and 90 weeks at the time of enrollment to ensure that children were not neonates and that all children would be younger than 60 months at the end of the intervention period. The age of children was verified, where possible, through birth and/or immunization records supplied by the caregiver and cross-checked by community health workers (health surveillance assistants [HSA]). The main caregiver of the child was selected as a study participant from each household.

### Data collection procedure.

A team of 10 enumerators were recruited and trained for 1 week before data collection. The enumerators were trained on study goals, practiced interview techniques, and translated the questionnaire into a local language (Chichewa). The training also included principles of human research subjects which ensured that human dignity, integrity, self-determination, rights, and confidentiality were safeguarded during the data collection process. One of the co-principal investigators supervised the data collection in the field throughout the baseline and follow-up surveys.

### Measures.

Face-to-face structured interviews, based on the RANAS model, were conducted with all participants to assess their self-reported handwashing and food hygiene practices. The questionnaire collected information about sociodemographic characteristics, food hygiene behaviors, psychosocial factors underlying food hygiene behaviors, hygiene proxy measures, and the recipient’s participation in the intervention (Table 1, Supplemental Annexes 1–3).

**Table 1:** Questions on targeted behaviors

Behaviors	Items	Answer format
Handwashing before eating main meals (e.g., lunch)	Before you feed your child main meals (e.g., lunch), how often do you wash your hands with soap and water?	(Almost) at no time–(almost) each time (5-point rating scale)
	Before your child takes main meals (e.g., lunch), how often does he/she wash hands with soap and water? (asked in case of child self-feeding)	
Handwashing after using the toilet	After you defecate, how often do you wash your hands with soap and water?	
Handwashing before food preparation	Before you prepare food, how often do you wash your hands with soap and water?	
Handwashing before eating snacks	Before you feed your child snacks, how often do you wash your hands with soap and water?	
	Before your child eats snacks, how often does he/she wash hands with soap and water? (asked in case of child self-feeding)	
Handwashing after cleaning child’s bottom	After cleaning child’s bottom, how often do you wash your hands with soap and water?	
Washing kitchen utensils with soap	Before you use kitchen utensils, how often do you wash them with soap and water?	(Almost) at no time–(almost) each time (5-point rating scale)
Keeping kitchen utensils on an elevated place	Do you keep your kitchen utensils on an elevated place?	Not at all–very much (5-point rating scale)
Reheating of leftover food	Do you reheat leftover food before being consumed?	Not at all–very much (5-point rating scale)
Feeding of child by the caregiver	Do you feed your child main meals (e.g., lunch and breakfast)?	Not at all–very much (5-point rating scale)

Response scales: 5-point rating scale (from “[almost] at no time” to “[almost] each time”; from “not at all” to “very much”).

### Behavior change intervention package.

Development of the intervention was derived from the formative research study conducted between February and July 2017 among 323 child caregivers in villages near and with similar characteristics to the study villages.^37,44^ The formative study identified different psychosocial factors for the targeted food hygiene behaviors to be included in an intervention. Thus, the intervention implemented different activities to address specific behavioral factors for each intervention package to facilitate improvement in targeted behaviors.

The complementary food hygiene behavior change intervention package that was implemented under the concept of “Hygienic Family” used cluster meetings and door-to-door household visits on alternating weeks, as the main communication channels^38^ because they have been proven to be effective in changing health behaviors.^45–47^ The concept of “Hygienic Family” aimed to promote the performance of the targeted behaviors by all family members. The interventions were facilitated by female community volunteers with support from government community health workers and Sanitation and Hygiene Applied Research for Equity (SHARE) project intervention staff. The community volunteers were trained for 2 days before implementing specific behavior change interventions in the cluster meetings. During door-to-door follow-up household visits, the community volunteers and HSAs reinforced the targeted behaviors that were discussed and demonstrated in the cluster meetings. Sanitation and Hygiene Applied Research for Equity project staff, who trained the community volunteers, conducted regular monitoring visits during cluster meetings and household follow-ups. Quarterly feedback meetings were conducted with community volunteers and HSAs to report on their performance, discuss lessons learned, and brainstorm solutions for any encountered challenges.

Implementation of the food hygiene package was conducted through two components, which included 1) handwashing with soap, where activities related to handwashing with soap were promoted through four cluster meetings and three household visits. The cluster meetings and household visits focused on the identified key handwashing behavior factors such as vulnerability, health knowledge, feelings, beliefs about costs and benefits, confidence in performance (provide infrastructure), others’ behavior, and remembering, which incorporated behavior change techniques (BCTs) of the RANAS model.^48^ 2) The food hygiene component implemented specific food hygiene activities through eight cluster meetings and seven household visits. Specifically, focal components were washing of kitchen utensils with soap, keeping the kitchen utensils in a safe (elevated) place, reheating of leftover food, and child feeding by the caregiver. For washing utensils with soap, the following behavior factors were included: health knowledge, others’ behavior, confidence in performance, and remembering. Keeping utensils in a safe place focused on health knowledge, costs and benefits, others’ behavior, confidence in performance, and remembering factors. Reheating of left-over food targeted behavior factors about feelings, others’ behavior, personal importance, and confidence in performance, whereas feeding of the child by the caregiver included others’ behavior, confidence in performance, and confidence in recovery. In total, these components of the intervention were implemented through 12 cluster meetings and 10 household visits. The cluster meetings took place at communal meeting places (e.g., church and village chief’s meeting ground) within targeted villages. Design of the intervention package was developed by SHARE project staff with support from the Department of Environmental Social Sciences at Eawag (Swiss Federal Institute of Aquatic Science and Technology) and the SHARE research advisory group that comprised sanitation and hygiene experts in Malawi. Training manuals are available on request, and description of the intervention package has been published elsewhere^38^ and briefly described in Supplemental Annex 4.

### Ethics.

Ethical approval for this study was received from the College of Medicine Research Ethics Committee (P.04/16/1935). The study was registered with the Pan-African Clinical Trials Registry (PACTR201703002084166). Written consent was received from all households willing to participate before allocation of a household identification number and associated barcode.

### Statistical analysis of data.

The statistical analysis of data was performed using IBM SPSS 23 Statistics software (IBM Corp., Armonk, NY), and the PROCESS macro for SPSS.^49^ Frequency analysis, ANOVAs, and *t*-test analysis methods were applied to answer our first and second research questions. The differences between baseline and follow-up data, and between the intervention and control groups were calculated for the targeted behaviors and the underlying psychosocial factors. Comparing the data from the baseline and follow-up surveys, and control and intervention groups revealed significant changes in targeted behaviors and changes in psychosocial factors. Mediation models were used to uncover underlying mechanisms and effects of an intervention on changes in target behaviors. Therefore, we computed a multiple mediation model using the PROCESS macro^49^ to answer our third research question. Only psychosocial factors with significant differences between the control and intervention groups were included in three separate multiple mediation models for each targeted behavior. We included intervention design (1 = intervention, 0 = control) as a predictor, changes in psychosocial factors as mediators, and changes in target behaviors as outcomes. The specific indirect (a*b), direct (c′), and total effects (c) of the predictor on outcomes were calculated.

## RESULTS

### Characteristics of the study population.

The analysis of respondent characteristics (*N* = 320) revealed that all participating caregivers were women, and the average household size was 5.30 (SD = 1.87). The majority of the caregivers were married (88%), and their average age was 28.6 years (SD = 8.6). Most participants (69.9%) had attended primary education, whereas 21.6% had not attended any formal education. All participating households had a child younger than 5 years whose average age was 32.1 months (SD = 6.1).

The monthly income of the respondents in Malawi Kwacha (MK) (1 USD= 740 MKW) varied greatly. It ranged from MK0 to MK9,999 among 34.1%, MK10,000 to MK19,999 among 24.1%, MK20,000 to MK29,999 among 18.4%, MK30,000 to MK39,999 among 14.4%, MK40,000 to MK49,000 among 5%, and over MK50,000 among 4.1%. The wealth index of the respondents included ownership of TV (2.2%), mobile phone (45.3%), electricity (4.4%), running water (1.3%), and bicycle (48.1%). The availability of soap in the households was observed in 86% of the households, and 73.1% of the respondents owned a farming area.

### Changes to targeted behaviors.

To answer our first research question, did target the behavior change because of the intervention, we compared differences in targeted behaviors between the intervention and control groups between baseline and follow-up surveys. As shown in Table 2, frequency analysis methods, *t*-tests, and ANOVAs were applied to answer the first question.

**Table 2:** Changes to target behaviors

	Control group (*N* = 80)			Intervention group (*N* = 240)			Intervention vs. control
Behavioral factors	M (SD) BL	M (SD) F	M (SD) diff*.* of mean *t*-test	M (SD) BL	M (SD) F	M (SD) diff*.* of mean *t*-test	Analysis of variance: diff*.* of mean *P*-value
Handwashing with soap at critical times	2.98 (1.21)	2.96 (1.00)	−0.02 (1.39)	2.91 (1.22)	4.41 (0.66)	1.49 (1.39)***	0.000
Washing kitchen utensils with soap	3.84 (1.34)	3.84 (1.08)	0.00 (1.57)	3.31 (1.46)	4.58 (0.68)	1.27 (1.64)***	0.000
Keeping kitchen utensils on an elevated place	2.85 (1.98)	2.23 (1.74)	−0.63 (2.48)*	2.08 (1.62)	4.57 (0.91)	2.49 (1.74)***	0.000
Reheating of leftover food	3.30 (1.31)	4.73 (0.67)	1.43 (1.47)***	3.74 (1.19)	4.67 (0.70)	0.93 (1.34)***	0.005
Feeding of child by the caregiver	2.33 (1.41)	2.83 (1.71)	0.50 (2.00)*	2.97 (1.46)	2.71 (1.71)	−0.26 (2.28)	0.008

BL = baseline; F = follow-up; diff = difference; M = mean; SD = standard deviation.

* *P* ≤ 0.05, ** *P* ≤ 0.01, *** *P* ≤ 0.001. Handwashing with soap at key times combined factors such as before eating, after using the toilet, after changing baby napkin, before preparing food, and before eating snack/fruit.

The statistical analysis, using *t*-test mean comparison, revealed significant differences in handwashing with soap, washing kitchen utensils with soap, keeping kitchen utensils in a safe place, and reheating of leftover food at the follow-up in the intervention group. However, there was a slight decrease in feeding children by the caregivers in the intervention group (Table 2). At the follow-up, a significant decrease in keeping kitchen utensils in a safe place was found in the control group. In addition, reheating of leftover food and feeding of children by the caregivers increased considerably among the control group. Nevertheless, there were no significant differences between baseline and follow-up in the control group for the handwashing with soap at key times and in washing kitchen utensils with soap. The ANOVA results showed a significant difference in differences between the intervention and control groups at follow-up in all the five targeted behaviors: handwashing with soap at key times, washing kitchen utensils with soap, keeping kitchen utensils in a safe place, reheating of left-over food, and feeding of children by the caregivers. However, the results for reheating of leftover food and feeding children by the caregivers changed significantly among the control group. As such, these two behaviors were not influenced by the intervention. Hence, only the other three significant targeted behaviors (i.e., handwashing with soap, washing kitchen utensils with soap, and keeping kitchen utensils in a safe place) were included for further analysis.

### Changes to the proxy measures about the targeted behaviors.

Statistical analysis (chi-square) revealed significant differences (*P* = 0.000) in differences between the intervention and control groups between baseline and follow-up surveys in all observed hygiene proxy factors: the presence of a handwashing facility (HWF), presence of soap and water at the HWF, presence of water and soap at the site where utensils were washed, and presence of a dish rack (Table 3). The presence of handwashing facilities and dish racks was observed in 95% and 96% of the participating households in the intervention group, respectively, at the end line compared with baseline (43% and 29%, respectively). And, 65% of the intervention households were observed to have water and soap at the dish-washing location, and 77% of the handwashing facilities had both soap and water. This indicated an increase from 31% to 20%, respectively, from what was observed at baseline. However, no significant changes were observed in the control group. Thus, the proxy measures conducted at baseline and follow-up surveys supported what was reported about the change in handwashing and utensil management behaviors among child caregivers in the intervention area.

**Table 3:** Changes in proxy measures

	Control (*N* = 79)			Intervention (*N* = 237)			Intervention vs. control
Proxy measures	BL, % (*n*)	F, % (*n*)	Diff., % (*n*)	BL, % (*n*)	F, % (*n*)	Diff., % (*n*)	Chi-square: diff. *P*-value
Presence of a HWF	51 (40)	35 (28)	−16 (−12)	43 (102)	95 (225)	52 (123)	0.000
Presence of soap and water at the HWF	24 (19)	18 (14)	−6 (−5)	20 (47)	77 (182)	57 (135)	0.000
Presence of soap and water at the utensil-washing location	28 (22)	24 (19)	−4 (−3)	31 (73)	65 (154)	34 (81)	0.000
Presence of a dish rack	39 (31)	26 (21)	−13 (−10)	29 (69)	96 (228)	67 (159)	0.000

BL = baseline; F = follow-up; diff = difference; HWF = handwashing facility.

* *P* ≤ 0.05, ** *P* ≤ 0.01, *** *P* ≤ 0.001.

### Changes in psychosocial factors underpinning behaviors such as handwashing with soap, washing kitchen utensils with soap, and keeping kitchen utensils in a safe place.

To answer our second research question, which psychosocial factors changed between the intervention and control groups, and how did these vary, we compared the differences in psychosocial factors underlying handwashing with soap, washing kitchen utensils with soap, and keeping kitchen utensils in a safe place between the intervention and control groups at baseline and follow-up surveys. We used frequency, *t*-test, and ANOVA mean comparison analysis methods (Tables 4–6).

**Table 4:** Differences in changes in risk, attitude, norms, ability, and self-regulation psychosocial factors explaining handwashing with soap between control and intervention groups

		Control group (*N* = 80)			Intervention group (*N* = 240)			Intervention vs. control group
Factor group	Behavioral factors	M (SD) BL	M (SD) F	M (SD) diff. of mean *t*-test	M (SD) BL	M (SD) F	M (SD) diff. of mean *t*-test	Analysis of variance: diff. of mean *P*-value
Risk factors	Vulnerability	1.68 (0.47)	3.24 (1.71)	1.56 (1.78)	1.75 (0.43)	2.84 (1.86)	1.09 (1.93)	0.054
	Severity	4.69 (0.8)	4.88 (0.43)	0.19 (0.8)	4.52 (1.09)	4.85 (0.55)	0.33 (1.19)	0.337
	Health knowledge	7.13 (2.32)	7.38 (1.85)	0.25 (2.88)	7.52 (2.62)	7.85 (2.01)	0.33 (3.47)	0.846
Attitude factors	Belief: effort	1.11 (0.64)	1.13 (0.51)	0.02 (0.77)	1.15 (0.66)	1.12 (0.51)	−0.03 (0.85)	0.669
	Belief: time consuming	1.08 (0.47)	1.14 (0.47)	0.06 (0.66)	1.13 (0.58)	1.12 (0.56)	−0.01 (0.83)	0.462
	Belief: expensive	1.84 (1.44)	1.98 (1.49)	0.14 (2.1)	1.73 (1.35)	1.58 (1.09)	−0.15 (1.83)	0.228
	Belief: certain prevention	4.59 (0.92)	4.35 (1.2)	−0.24 (1.54)	4.63 (0.84)	4.67 (0.88)	0.04 (1.20)	0.112
	Feelings (like)	3.66 (1.35)	3.71 (1.17)	0.05 (1.69)	3.58 (1.39)	4.61 (0.76)	1.03 (1.49)	0.000***
Norm factors	Others’ behavior in the household	3.75 (1.38)	3.18 (1.34)	−0.57 (1.89)	3.14 (1.47)	4.28 (1.05)	1.14 (1.82)	0.000***
	Others’ behavior in the village	2.78 (1.06)	3.18 (1.34)	0.4 (1.63)	2.52 (0.98)	4.28 (1.05)	1.76 (1.45)	0.000***
	Others approval	4.68 (0.76)	4.36 (1.14)	−0.32 (1.36)	4.52 (0.96)	4.77 (0.66)	0.25 (1.17)	0.000***
Ability factors	Confidence in performance	4.43 (1.12)	4.06 (1.14)	−0.37 (1.68)	3.99 (1.44)	4.69 (0.68)	0.70 (1.56)	0.000***
	Difficulty getting water	1.05 (0.27)	1.08 (0.38)	0.03 (0.45)	1.13 (0.64)	1.23 (0.78)	0.10 (1.03)	0.548
	Difficulty getting soap	2.09 (1.45)	2.23 (1.28)	0.14 (1.9)	2.02 (1.51)	1.69 (1.04)	−0.33 (1.85)	0.048*
	Difficulty getting time	1.25 (0.77)	1.13 (0.56)	−0.12 (0.75)	1.25 (0.82)	1.20 (0.0.70)	−0.05 (1.08)	0.566
	Barrier: distance	4.13 (1.4)	3.91 (1.21)	−0.22 (1.94)	3.74 (1.53)	4.61 (0.86)	0.87 (1.75)	0.000***
Self-regulation factors	Remembering (pay attention)	3.78 (1.54)	3.91 (1.06)	0.13 (1.88)	3.36 (1.57)	4.59 (0.80)	1.23 (1.68)	0.000***
	Remembering (forgetting last 24 hours)	2.00 (1.30)	2.51 (1.51)	0.51 (1.94)	2.38 (1.51)	1.44 (0.99)	−0.94 (1.80)	0.000***
	Commitment (important)	4.88 (0.49)	4.68 (0.88)	−0.20 (1.05)	4.85 (0.54)	4.82 (0.63)	−0.03 (0.84)	0.132
	Commitment (commitment)	4.63 (0.85)	4.19 (1.08)	−0.44 (1.38)	4.48 (1.05)	4.82 (0.52)	0.34 (1.20)	0.000***

BL = baseline; F = follow-up; diff. = difference.

* *P* ≤ 0.05, ** *P* ≤ 0.01, *** *P* ≤ 0.001. All questions included 5-point rating scales and response choices from “1 = not at all” to “5 = very much.” Health knowledge: sum scale (0–13).

**Table 5:** Differences in changes in risk, attitude, norms, ability, and self-regulation psychosocial factors explaining washing kitchen utensils with soap between control and intervention groups

		Control group (*N* = 80)			Intervention group (*N* = 240)			Intervention vs. control group
Factor group	Behavioral factors	M (SD) BL	M (SD) F	M (SD) diff. of mean	M (SD) BL	M (SD) F	M (SD) diff. of mean	Analysis of variance: diff. of mean *P*-value
Risk factors	Vulnerability	1.68 (0.47)	3.24 (1.71)	1.56 (1.77)	1.75 (0.43)	2.84 (1.86)	1.09 (1.93)	0.054
	Severity	4.69 (0.80)	4.88 (0.43)	0.19 (0.79)	4.52 (1.09)	4.85 (0.0.55)	0.33 (1.19)	0.337
	Health knowledge	7.13 (2.32)	7.38 (1.85)	0.25 (2.88)	7.52 (2.62)	7.85 (2.01)	0.33 (3.47)	0.846
Attitude factors	Belief: effort	1.11 (0.50)	1.13 (0.54)	0.02 (0.75)	1.13 (0.50)	1.14 (0.55)	0.01 (0.75)	0.932
	Belief: time consuming	1.23 (0.69)	1.2 (0.62)	−0.03 (0.93)	1.22 (0.65)	1.23 (0.75)	0.01 (1.03)	0.797
	Belief: pleasant	4.79 (0.72)	4.65 (0.96)	−0.14 (1.11)	4.50 (1.03)	4.63 (1.04)	0.13 (1.39)	0.133
Norm factors	Others’ behavior in the household	3.24 (1.33)	3.23 (0.98)	−0.01 (1.56)	2.74 (1.16)	3.73 (1.01)	0.99 (1.49)	0.000***
	Others’ behavior in the village	3.19 (0.99)	3.24 (0.89)	0.05 (1.17)	2.55 (0.90)	3.53 (0.84)	0.98 (1.16)	0.000***
	Others’ approval	3.55 (1.73)	3.73 (1.58)	0.18 (2.18)	3.72 (1.63)	4.29 (1.18)	0.57 (2.00)	0.132
	Personal obligation	2.54 (1.82)	3.35 (1.68)	0.81 (2.17)	2.35 (1.78)	3.43 (1.75)	1.08 (2.29)	0.354
Ability factors	Confidence in performance	4.25 (1.42)	3.83 (1.27)	−0.42 (1.98)	3.64 (1.60)	4.60 (0.83)	0.96 (1.66)	0.000***
	Difficulty getting water	4.08 (1.50)	4.08 (1.27)	0.00 (1.92)	3.7 (1.53)	4.68 (0.76)	0.98 (1.66)	0.000***
	Difficulty getting soap	2.74 (1.06)	2.58 (1.21)	−0.16 (1.50)	2.92 (1.21)	1.74 (0.90)	−1.18 (1.90)	0.000***
	Confidence in performance (recovery)	4.56 (0.93)	4.23 (1.03)	−0.33* (1.48)	4.05 (1.37)	4.69 (0.73)	0.64 (1.52)	0.000***
Self-regulation factors	Remembering (pay attention)	3.55 (1.73)	3.9 (1.19)	0.35 (1.90)	3.72 (1.63)	4.62 (0.73)	0.90 (1.75)	0.018*
	Remembering (forgetting last 24 hours)	3.95 (1.35)	1.58 (1.18)	−2.38 (1.86)	3.45 (1.51)	1.36 (0.89)	−2.09 (1.74)	0.210
	Commitment (importance)	4.74 (0.84)	4.74 (0.74)	0.00 (1.0)	4.86 (0.54)	4.83 (0.54)	−0.03 (0.79)	0.732
	Commitment (commitment)	3.98 (1.56)	4.39 (0.99)	0.41 (1.91)	3.66 (1.69)	4.72 (0.70)	1.06 (1.78)	0.006**

BL = baseline; F = follow-up; diff = difference.

* *P* ≤ 0.05, ** *P* ≤ 0.01, *** *P* ≤ 0.001. All questions included 5-point rating scales and response choices from “1 = not at all” to “5 = very much.” Health knowledge: sum scale (0–13).

**Table 6:** Differences in changes in risk, attitude, norms, ability, and self-regulation psychosocial factors explaining keeping kitchen utensils in a safe place between control and intervention groups

		Control group (*N* = 80)			Intervention group (*N* = 240)			Intervention vs. control
Factor group	Behavioral factors	M (SD) BL	M (SD) F	M (SD) diff. of mean	M (SD) BL	M (SD) F	M (SD) diff. of mean	analysis of variance: diff. of mean *P*-value
Risk factors	Vulnerability	1.68 (0.47)	3.24 (1.71)	1.56 (1.77)	1.75 (0.43)	2.84 (1.86)	1.09 (1.93)	0.054
	Severity	4.69 (0.80)	4.88 (0.43)	0.19 (0.79)	4.52 (1.09)	4.85 (0.0.55)	0.33 (1.19)	0.337
	Health knowledge	7.13 (2.32)	7.38 (1.85)	0.25 (2.88)	7.52 (2.62)	7.85 (2.01)	0.33 (3.47)	0.846
Attitude factors	Belief: effort	1.1 (0.52)	1.14 (0.49)	0.04 (0.74)	1.19 (0.0.65)	1.10 (0.49)	−0.09 (0.81)	0.207
	Belief: time consuming	1.1 (0.34)	1.23 (0.78)	0.13 (0.86)	1.28 (0.82)	1.20 (0.75)	−0.08 (1.13)	0.149
	Belief: pleasant	4.44 (1.04)	4.53 (1.02)	0.09 (1.45)	4.44 (1.18)	4.69 (0.90)	0.25 (1.52)	0.403
Norm factors	Others’ behavior in the household	4.08 (1.33)	4.15 (1.19)	0.07 (1.87)	4.12 (1.29)	4.58 (0.79)	0.46 (1.50)	0.059
	Others’ behavior in the village	2.68 (1.11)	2.48 (0.89)	−0.2 (1.36)	2.30 (0.81)	3.33 (0.84)	1.03 (1.12)	0.000***
	Others’ approval	4.01 (1.46)	3.30 (1.56)	−0.71 (2.094)	3.86 (1.53)	4.25 (1.23)	0.39 (1.91)	0.000***
	Personal obligation	2.56 (1.81)	2.99 (1.66)	0.43 (2.18)	2.41 (1.78)	3.54 (1.73)	1.13 (2.55)	0.028*
Ability factors	Confidence in performance (hurry)	3.88 (1.62)	3.83 (1.27)	−0.05 (2.11)	3.38 (1.71)	4.60 (0.83)	1.22 (1.82)	0.000***
	Confidence in performance (no place)	4.03 (1.41)	3.98 (1.21)	−0.05 (1.88)	3.71 (1.56)	4.7 (0.73)	0.99 (1.70)	0.000***
	Confidence in performance (cannot do)	2.61 (1.48)	3.25 (1.61)	0.64 (2.29)	2.31 (1.06)	1.52 (0.94)	−0.79 (1.49)	0.000***
	Confidence in performance (recovery)	4.29 (1.29)	3.95 (1.26)	−0.34 (0.00)	3.95 (1.47)	4.63 (0.73)	0.68 (0.00)	0.000***
Self-regulation factors	Remembering (pay attention)	2.61 (1.82)	2.95 (1.71)	0.34 (2.50)	2.75 (1.75)	4.72 (0.74)	1.97 (1.93)	0.000***
	Remembering (forgetting last 24 hours)	2.63 (1.37)	2.76 (1.78)	0.13 (2.30)	2.42 (1.18)	1.38 (0.95)	−1.04 (1.47)	0.000***
	Commitment (importance)	4.73 (0.76)	4.66 (0.79)	−0.07 (1.12	4.61 (0.92)	4.88 (0.46)	0.27 (1.03)	0.015*
	Commitment (commitment)	3.36 (1.79)	4.3 (1.06)	0.94 (2.24)	3.56 (1.76)	4.84 (0.50)	1.28 (1.81)	0.175

diff. = difference; BL = baseline; F = follow up.

* *P* ≤ 0.05, ** *P* ≤ 0.01, *** *P* ≤ 0.001. All questions included 5-point rating scales and response choices from “1 = not at all” to “5 = very much.” Health knowledge: sum scale (0–13).

Changes in psychosocial factors underlying handwashing identified 10 factors with significant differences between the control and intervention groups. Analysis of variance revealed feelings, others’ behavior in the household, others’ behavior in the village, others’ approval, confidence in performance, difficulty to get enough soap for handwashing, distance as a barrier, remembering (pay attention), remembering (forgetting last 24 hours), and commitment as significant factors for the handwashing with soap behavior (Table 4). These significant factors were included in the mediation model as mediators.

The results for changes in psychosocial factors underlying washing kitchen utensils with soap revealed eight factors with a significant difference in differences between the control and intervention groups. As shown in Table 5, ANOVA revealed the following significant factors: others’ behavior in the household, others’ behavior in the village, confidence in performance, difficulty to get enough water, difficulty to get enough soap, confidence in performance (recovery), remembering (pay attention), and commitment. Again, these changes in psychosocial factors were included for further mediation analysis.

For keeping kitchen utensils in a safe place, 10 factors were identified with a significant difference in differences between the control and intervention groups at the follow-up. According to ANOVA results, behavioral factors such as others’ behavior in the village, others’ approval, personal obligation, confidence in performance (hurry), confidence in performance (no place), confidence in performance (cannot do), confidence in performance (recovery), remembering (pay attention), remembering (forgetting last 24 hours), and commitment (importance) were significant for the behavior of keeping kitchen utensils in a safe place (Table 6). Thus, these significant factors were included for further multiple mediation analysis.

### Changes in psychosocial factors as mediators.

**Figure 1.**
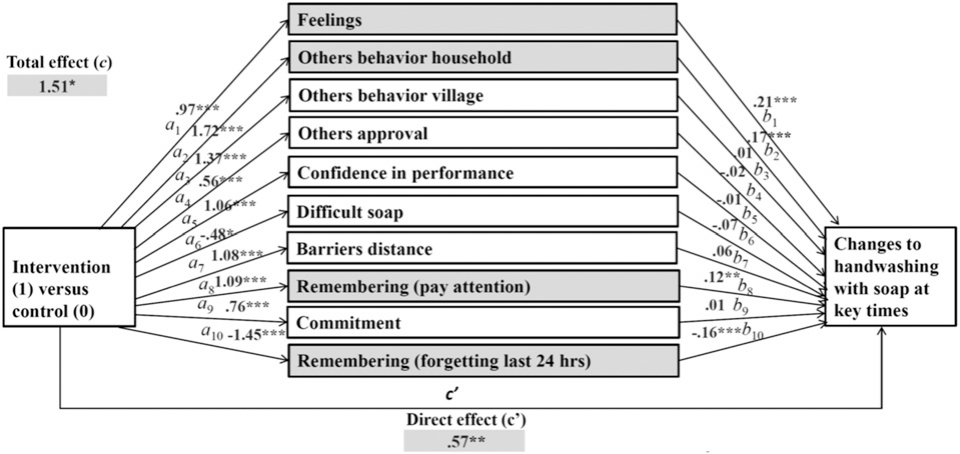
Multiple mediation: effects of intervention on changes to handwashing with soap via changes in psychosocial factors (mediators).

**Figure 2.**
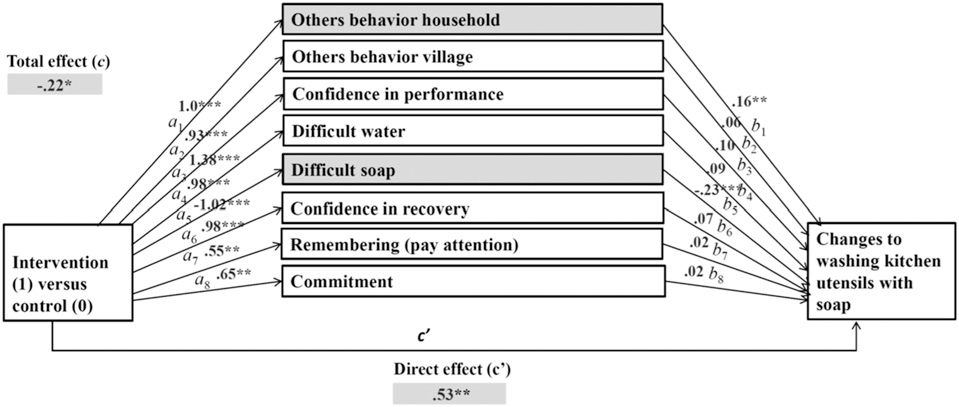
Multiple mediation: effects of intervention on changes to washing kitchen utensils with soap via changes in psychosocial factors (mediators).

**Figure 3.**
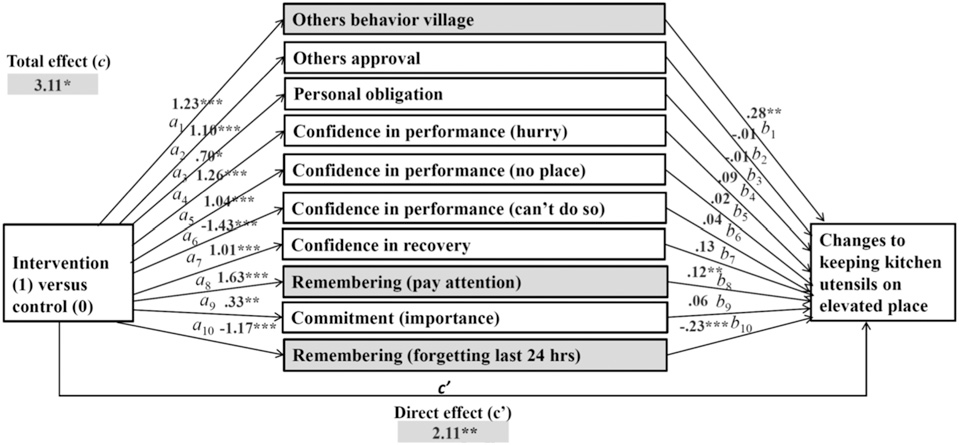
Multiple mediation: effects of intervention on changes to keeping kitchen utensils at a safe place with soap via changes in psychosocial factors (mediators).

To answer our third research question, which psychosocial factors changed because of the intervention and, therefore, changed the behaviors, three multiple mediations were computed for the behaviors of handwashing with soap, washing kitchen utensils with soap, and keeping kitchen utensils in a safe place. In our multiple mediation models, intervention (yes/no) was included as predictors, changes in psychosocial factors as mediators, and changes in the target behavior as outcomes. Only factors with a significant difference in differences between the control and intervention groups were selected for mediation analysis as shown in Figures 1–3. Our calculations included specific indirect (a*b), direct (c′), and total effects (c) of the intervention on changes to targeted behaviors. The specific indirect effects (a*b) are defined as the effects of the intervention (predictor X) via psychosocial factors (mediators M) on targeted behaviors (outcome Y). The direct effect (c′) is defined as the effect of intervention on changes to targeted behaviors when all mediators are included in the model. The total effects (c) include all factors calculated in the mediation model.

Our findings from multiple mediation calculations suggest significant specific indirect effects of the intervention on handwashing with soap in the following four psychosocial factors (factors marked gray in Figure 1): feelings (*b =* 0.2049, [CI: 0.0990–0.3458]), others’ behavior household (*b* = 0.2850, [CI: 0.1120–0.4854]), remembering (pay attention) (*b* = 0.1304, [CI: 0.0366–0.2530]), and remembering (forgetting last 24 hours) (*b* = 0.2337, [CI: 0.1112–0.3794]). That is, these factors mediated the relationship between intervention and changes in handwashing with soap at key times.

Factors such as others’ behavior in the village, others’ approval or disapproval, confidence in performance, difficulty to get enough soap, distance as a barrier, and commitment did not explain handwashing (path “b” not significant). Thus, though these factors did not bring any significant change to the behavior, they were influenced by the intervention (path “a” significant).

Multiple mediation models for the effects of the intervention on changes in washing kitchen utensils with soap revealed significant specific indirect effects in two psychosocial factors (factors marked grey in Figure 2): others’ behavior household (*b =* 0.1574, [CI: 0.0461–0.3019]) and difficulty in having enough soap (*b* = 0.2367, [CI: 0.1038–0.3986]). Meaning that, these factors mediated the effects of the intervention on washing kitchen utensils with soap behavior.

Psychosocial factors such as others’ behavior in the village, confidence in performance, difficulty in having enough water, confidence in performance (recovery), remembering (paying attention), and commitment were not predictors of washing kitchen utensils with soap (path “b” not significant). However, as shown in path “a,” the intervention also significantly influenced these factors, despite being irrelevant in changing the behavior (path “a” significant).

Multiple mediation analysis results for the effects of the intervention on changes in keeping kitchen utensils in a safe place revealed significant specific indirect effects in three psychosocial factors (factors marked gray in Figure 3): others’ behavior in the village (*b =* 0.3507, [CI: 0.0825–0.6260]), remembering (paying attention) (*b* = 0.1962, [CI: 0.0349–0.3878]), and remembering (forgetting last 24 hours) (*b* = 0.2635, [CI: 0.0853–0.4685]). Thus, these factors were mediators on the effects of the intervention on keeping kitchen utensils in a safe place.

Psychosocial factors including others’ approval, personal obligation, confidence in performance (hurry), confidence in performance (no place), confidence in performance (cannot do), confidence in performance (recovery), and commitment (importance) did not explain keeping kitchen utensils in a safe place behavior (path “b” not significant). Hence, the intervention influenced these psychosocial factors. However, they were not relevant in changing the behavior (path “a” significant).

## DISCUSSION

### Interpretation of study results.

This study investigated the effectiveness of an intervention package derived from evidence-based data using the RANAS model of the behavior change^32,48^ that aimed to improve complementary food hygiene practices in rural Malawi. The evidence-based interventions targeted the following food hygiene behaviors: handwashing with soap at key times, washing kitchen utensils with soap, keeping kitchen utensils in a safe place, reheating of leftover food, and feeding of children by caregivers. This study aimed to identify the underlying mechanisms of the intervention on target behaviors using the multiple mediation analysis method^49^ to identify which interventions were most effective in changing the behaviors. The study results have shown that most households in the study setting live below the World Bank’s extreme poverty line of USD 1.90 a day,^50^ a situation that requires further attention and context-appropriate health promotion strategies.

The results of the study for the first research question, did complementary food hygiene behaviors change because of the intervention, suggest a significant increase in three target behaviors after the intervention: handwashing with soap, washing kitchen utensils with soap, and keeping kitchen utensils in a safe place. These results confirmed the effectiveness of the RANAS model in developing and testing evidence-based interventions for food hygiene behaviors, the first of its kind. These findings are also in line with previous research examining the effects of behavior change interventions on hygiene, for example, handwashing with soap.^51–53^ On the proxy measures (availability of an HWF, availability of soap and water at the HWF, and availability of an elevated place for keeping kitchen utensils), the study results showed a significant increase after intervention implementation in the treatment group. This increase in WASH infrastructure was as a result of promotion activities that encouraged the caregivers to install the facilities. Previous research suggests that availability of infrastructure is a strong predictor for successful performance of desired target behaviors.^54^

The study results for the second research question, which psychosocial factors vary between the intervention and control groups, revealed a significant difference in differences between the intervention and control groups at the time of follow-up survey. These factors were included in further mediation models to investigate the most effective interventions and to uncover underlying mechanisms of the effects on targeted food hygiene behaviors via psychosocial factors.

Establishing a relationship between the intervention and changes in the targeted behavior does not translate to an understanding of exactly how interventions affect the behavior change.^49^ As such, mediation models can be used to uncover underlying mechanisms of the evidence-based behavior change in the public health sector.^36,52,55^ The results of this mediation analysis indicated that some changes in psychosocial factors were mediators of the improved changes noticed in the targeted food hygiene behaviors.

For changes in handwashing practice, mediation analysis uncovered feelings, others’ behavior in the household, remembering (pay attention), and remembering (forgetting last 24 hours) as significant mediators. This, in turn, confirms the effectiveness of the behavior change intervention elements targeting, first, feelings (BCT 8) by describing feelings about performing and consequences of handwashing without soap; second, others’ behavior in the household (BCT 9) by encouraging that others already perform the behavior; and third, remembering (BCT 34) by using memory aids and environmental prompts. The remaining factors in the mediation analysis were included in the intervention, but had no significant influence on the behavior. In summary, the cues for action increased the ability of the child caregivers to wash their hands with soap. In addition, the intervention significantly increased their positive feelings (like) about handwashing with soap. It also increased the perception by caregivers that other household members also performed handwashing with soap, which in return increased the caregivers practice. This adds to the growing research indicating a need to incorporate psychosocial factors, in addition to contextual elements, for the success of handwashing with soap promotion interventions.^54,56,57^

For intervention effects on changes in washing kitchen utensils with soap, significant mediators were changes in others’ behavior in the household, targeted by encouraging others that some are already performing the behavior (BCT 9), and changes in the difficulty of having enough soap to wash kitchen utensils (“demonstrate and model behavior”, BCT 17), which targeted ability, for example, confidence in performance. This is again a confirmation of the effectiveness of the tested interventions. Other remaining factors included in the mediation were tackled by the intervention, but exerted no significant influence on the behavior. In summary, the intervention significantly increased the influence of others’ behavior in the household among study participants. Furthermore, the intervention increased the participants’ understanding on the importance of using soap when washing utensils. This enabled them to prioritize soap for utensil washing (i.e., became less difficult to have soap), and this subsequently increased the performance of the behavior.

The mediators that influenced changes to the behavior about keeping utensils in a safe place included others’ behavior in the village, remembering (pay attention), and remembering (last 24 hours). This confirms the effectiveness of incorporating public commitment (BCT 10), memory aids, and environmental prompts (BCT 34) in the intervention. Other factors included in the model were influenced by the intervention, but were not found to be significant for the behavior change. In summary, the intervention significantly increased the influence of the behavior of others in the village and remembering to keep kitchen utensils in a safe place among study participants, which in turn increased the practice.

Finally, the intervention package in our study included multiple BCT’s that were derived from evidence-based baseline data.^37^ Previous health behavior change research suggests that multiple behavior change interventions could provoke coaction,^58^ which in turn increases the effectiveness of the whole intervention package. However, recent studies from Bangladesh and Kenya focused on WASH, and nutrition behaviors showed no differences between single and multiple interventions.^59,60^ Despite the increase in targeted behaviors, some interventions from our study changed specific psychosocial factors significantly which, however, had no impact in changing the behavior. These findings are helpful to refine the intervention package. The BCTs corresponding to the significant psychosocial factors that were not relevant in changing the targeted behaviors could be further reviewed in future research interventions.

In summary, findings from our research study revealed a significant increase in self-reported target behaviors and in behavioral proxies after the intervention, uncovered the underlying mechanisms of behavior change interventions on target behaviors, and showed which interventions, (e.g., BCT’s from the RANAS catalogue) were most effective in changing the behaviors. This research is especially relevant for future projects to refine behavior change interventions in this particular population and to ensure time and resources target interventions with the best opportunity for success.

### Practical implications.

The study results provide a platform and an opportunity to further integrate food hygiene into WASH and nutrition programming. In addition, the identified handwashing with soap behavior factors could be used to promote handwashing in existing sanitation programs such as community-led total sanitation to maintain a sustained behavior change. As such, our evidence-based research study is important for policy makers and programming in a number of ways.

First, community volunteers from the intervention area can be identified to deliver the behavior-centered intervention successfully. This process could, therefore, be integrated with existing programs such as scaling up nutrition caregiver groups and village health committees. However, community health workers must be available to regularly backstop the services of community volunteers with their expertise. Thereafter, handwashing BCTs from the intervention, addressing feelings, others’ behavior in the household, and remembering should be practically delivered to the caregivers in conjunction with facts about the link between the handwashing practice and onset of diarrheal diseases.

Second, BCTs for washing utensils with soap targeting others’ behavior in the household and difficulty to get enough soap should be implemented. Thus, the effective use of the intervention may encourage households to realign priorities for soap, which is critical in such low-income settings. For continuity, key handwashing with soap messages initially introduced should be incorporated and reiterated during this process.

Third, the caregivers should be introduced to the concept of keeping utensils in a safe place that will focus on others’ behavior in the village and remembering. Importantly, already delivered behaviors (i.e., handwashing with soap and washing utensils with soap) need to be integrated in the implementation of this behavior. In addition, to foster confidence in performance, the demonstration on how to construct their own handwashing facilities and dish racks should be repeated from time to time among the child caregivers.

The perception of how others behave (others’ behavior) had a strong significance across all three behaviors. Thus, a strong emphasis on these normative elements through the intervention may be necessary to successfully promote the desired food hygiene behaviors. In addition, this has demonstrated the importance of using the concept of “Hygienic Family” to influence the behavior of all family members.

By refining the interventions using psychological theories (e.g., the RANAS model) and specific statistical analysis methods (e.g., mediation analysis), the study has shown the effectiveness of incorporating the significant behaviors in the promotion of complementary food hygiene practices in rural household settings.

### Limitations.

Self-reported health behaviors are prone to bias.^61^ However, this was controlled by conducting spot checks on a number of variables (i.e., handwashing with soap, washing utensils with soap, and keeping utensils in a safe place) that were reported by the participants. Much as the government extension health workers HSAs were incorporated in the delivery of the intervention, their participation (i.e., supervising the volunteers) was affected by their high workload because they are responsible for all health-related activities at the community level. However, this was addressed by using field intervention supervisors. Nevertheless, this may have an implication on the long-term sustainability and scalability of the intervention because the hired field intervention supervisors would not be there when the research project comes to an end. As such, there is a need for a follow-up study to assess how the existing structures have continued with the interventions without external support. Although the sample size in this study was less than the ANOVA calculation required, we are confident that the significant differences in behaviors reported reflect the legitimate impact of the intervention. Nevertheless, further data collection would support validation. The use of mass media as a communication channel should be taken into consideration in promoting the key behaviors on a wider scale.

## CONCLUSION

The research study in this article is the first to address food hygiene behaviors using the RANAS behavior change approach. The evidence-based interventions successfully changed handwashing, washing utensils with soap, and keeping utensils in a safe place among the intervention households. In addition, our research study uncovered underlying behavior change mechanisms by identifying specific psychosocial factors relevant in changing the behaviors. However, further research should test other potential mediators or moderators of behavior.

Thus, the intervention package used in our study can be recommended for promotion of the behavior change to handwashing with soap, washing of utensils with soap, and keeping utensils in a safe place in rural settings of Malawi.

## Supplemental annexes

Supplemental materials
