# Supplementary material for: Improving Complementary Food Hygiene Behaviors Using the Risk, Attitude, Norms, Ability, and Self-Regulation Approach in Rural Malawi
Source: Am J Trop Med Hyg. 2020 Feb 24;102(5):1104–15. doi: 10.4269/ajtmh.19-0528 (PMC7204602; doi:10.4269/ajtmh.19-0528)
Supplement: Supplementary file 1 [file tpmd190528.SD1.pdf]

Supplemental Annex 1: Questionnaire based on the RANAS model for handwashing with soap behavior

Behavior Determinants	Selected Items
Risk Factors	
Vulnerability	In general, how high do you think is the risk that you get diarrhea?
Severity	Imagine that you contracted diarrhea how severe would be the impact on your life in general?
Health Knowledge	Can you tell me what causes diarrhea? Could you please tell me for each following aspects whether it is a cause or not? E.g. Water contaminated by bacteria.
Attitudinal Factors	
Belief effort	How effortful do you think is washing hands with soap and water?
Belief time consuming	How time consuming do you think it is to always wash hands with soap and water?
Belief expensive	How expensive is it for you to always wash hands with soap and water?
Belief distance (far away)	Do you think that the hand washing facility is far away from your usual area of activity?
Belief certain for prevention	How certain are you that always washing hands with soap and water prevents you and your family from getting diarrhea?
Feelings	How much do you like always washing hands with soap and water?
Normative Factors	
Others' behavior household	How many people of your household always wash hands with soap and water?
Others' behavior village	How many people of your village always wash hands with soap and water?
Others' approval	People who are important to you like your family members, friends, the chief of the village, NGO workers or Pastor, how much they approve that you always wash hands with soap and water?
Personal obligation	How strong do you feel a personal obligation to yourself to wash hands with soap and water?
Ability Factors	
Confidence in performance	How sure are you that you can wash hands with soap and water?
Difficult water	How difficult is to get as much water as you need to always wash hands with soap and water?
Difficult soap	How difficult is to get much soap as you need to always wash hands with soap and water?
Difficult time	How difficult is it have enough time to always wash hands with soap and water
Barriers distance	How confident are you that you can wash hands with soap and water, even if you have to walk some distance to reach the next hand washing facility?
Self-Regulation Factors	
Coping plan	Do you have a plan what to do so that you always have soap for hand washing? Please specify.
Remembering (pay attention)	How much do you pay attention to always have enough soap at home to wash hands with soap and water?
Remembering (forgetting last 24h)	When you think about the last 24 hours: How often did it happen that you forgot to wash your hands with soap and water?
Commitment (important)	How important is it for you to wash hands with soap and water?

Additional factor	How often do you discuss with others about handwashing with soap at critical times
Self-reported Behavior	
Hand washing before eating main meals (e.g. lunch)	Before you feed your child main meals (e.g. lunch), how often do you wash your hands with soap and water?<br>Before your child takes main meals (e.g. lunch), how often does he/she wash hands with soap and water? *(asked in case of child self-feeding)*
Hand washing after using the toilet	After you defecate, how often do you wash your hands with soap and water?
Hand washing before food preparation	Before you prepare food, how often do you wash your hands with soap and water?
Hand washing before eating snacks	Before you feed your child snacks, how often do you wash your hands with soap and water?<br>Before your child eat snacks, how often does he/she wash hands with soap and water? *(asked in case of child self-feeding)*
Hand washing after cleaning child's bottom	After cleaning child's bottom, how often do you wash your hands with soap and water

Notes. Response scales: 5-point Likert scale [from 'not at all' to 'very much'; from 'at no time' to 'almost each time'; from 'never' to 'very often'; from 'nobody' to 'almost all of them'], [yes; no; I don't know].

Supplemental Annex 2: Questionnaire based on the RANAS model for washing kitchen utensils with soap behavior

**Behavior Determinants**	**Selected Items**
Risk Factors	
Vulnerability	In general, how high do you think is the risk that you get diarrhea?
Attitudinal Factors	
Belief effort	How effortful do you think is washing utensils with soap and water?
Belief time consuming	How time consuming do you think it is to always wash utensils with soap and water?
Belief pleasant	How pleasant is it for you to always wash utensils with soap and water?
Normative Factors	
Others' behavior household	How many people of your household always wash utensils with soap and water?
Others' behavior village	How many people of your village always wash utensils with soap and water?
Others' approval	People who are important to you like your family members, friends, the chief of the village, NGO workers or Pastor, how much they approve that you always wash utensils with soap and water?
Personal obligation	How strong do you feel a personal obligation to yourself to wash utensils with soap and water?
Ability Factors	
Confidence in performance	How sure are you that you can wash utensils with soap and water?
Difficult water	How difficult is to get as much water as you need to always wash utensils with soap and water?
Difficult soap	How difficult is to get much soap as you need to always wash utensils with soap and water?
Confidence in performance (hurry)	How sure are you that you can always wash utensils with soap even if you are in hurry?

Confidence in performance (recovery)	If you stopped washing utensils with soap for other reasons, how sure are you that you can restart washing utensils with soap and water?
Self-Regulation Factors	
Remembering (pay attention)	How much do you pay attention to always have enough soap at home to wash utensils with soap and water?
Remembering (forgetting last 24h)	When you think about the last 24 hours: How often did it happen that you forgot to wash utensils with soap and water?
Commitment (important)	How important is it for you to wash utensils with soap and water?
Commitment (committed)	How committed do you feel to wash utensils with soap and water?
Additional factor	How often do you discuss with others about washing utensils with soap and water
Self-reported Behavior	
Washing kitchen utensils with soap	Before you use kitchen utensils, how often do you wash them with soap and water

Notes. Response scales: 5-point Likert scale [from 'not at all' to 'very much'; from 'at no time' to 'almost each time'; from 'never' to 'very often'; from 'nobody' to 'almost all of them'].

Supplemental Annex 3: Questionnaire based on the RANAS model for keeping kitchen utensils on safe place behavior

**Behavior Determinants**	**Selected Items**
Risk Factors	
Vulnerability	In general, how high do you think is the risk that you get diarrhea?
Attitudinal Factors	
Belief effort	How effortful do you think is to keep utensils on an elevated place?
Belief time consuming	How time consuming do you think it is to always keep utensils on an elevated place?
Belief pleasant	How pleasant is it for you to always keep utensils on an elevated place?
Normative Factors	
Others' behavior household	How many people of your household always keep utensils on an elevated place?
Others' behavior village	How many people of your village always keep utensils on an elevated place?
Others' approval	People who are important to you like your family members, friends, the chief of the village, NGO workers or Pastor, how much they approve that you always keep utensils on an elevated place?
Personal obligation	How strong do you feel a personal obligation to yourself to keep utensils on an elevated place?
Ability Factors	
Confidence in performance (no place)	How confident are you that you can keep kitchen utensils on an elevated place even if this is difficult sometimes (e.g. because of no dish rack)?
Confidence in performance (hurry)	How sure are you that you can always keep utensils on an elevated place even if you are in hurry?
Confidence in performance (recovery)	If you stopped washing utensils with soap for other reasons, how sure are you

Confidence in performance (can't do so)	that you can restart keeping utensils on an elevated place?<br><br>How often does it happen that you want to keep kitchen utensils on an elevated position but can't do so?
Self-Regulation Factors	
Remembering (pay attention)	How much do you pay attention to always keep utensils on an elevated place?
Remembering (forgetting last 24h)	When you think about the last 24 hours: How often did it happen that you forgot to keep utensils on an elevated place?
Commitment (important)	How important is it for you to keep utensils on an elevated place?
Commitment (committed)	How committed do you feel to keep utensils on an elevated place?
Additional factor	How often do you discuss with others about keeping utensils on an elevated place
Self-reported Behavior	
Keeping kitchen utensils safe (on elevated place)	Do you keep your kitchen utensils on an elevated place

Notes. Response scales: 5-point Likert scale [from 'not at all' to 'very much'; from 'at no time' to 'almost each time'; from 'never' to 'very often'; from 'nobody' to 'almost all of them'].

Supplemental Annex 4. Description of intervention package.

Handwashing with soap behavior change intervention elements

The first element covered a paint - game disease spread exercise and glo-germ practical demonstration which were implemented to demonstrate faecal oral route disease transmission and the effectiveness of handwashing with soap in removing germs. This activity covered several aspects of handwashing that targeted vulnerability, health knowledge and feelings factors which incorporated Behavior Change Techniques (BCTs) 1, 2, 3 and 8.[41]

The second element presented five handwashing with soap critical times (i.e. handwashing with soap after changing baby nappy, after using latrine, before food preparation, before eating snack/fruit and before child feeding/eating). This was emphasized through use of poster presentations in cluster meetings where the recipients discussed about "what" and "why" the four critical times that stimulated an interesting conversation. Use of posters encouraged the

caregivers to talk amongst themselves about the behavior. This element focused on factors health knowledge and beliefs about costs and benefits (BCTs 1, present facts and 7, beliefs about costs and benefits).

The third element targeted factor confidence in performance (BCT 16, ‘provide infrastructure’) which prompted and supported the targeted households to construct handwashing facilities (tippy-tap). In addition, it strengthened caregivers’ ability to perform the behavior since the community volunteers demonstrated to the caregivers on the recommended steps to properly wash hands with soap (BCT 18, “prompt guided practice”). This activity also proved to others that some caregivers already have handwashing facilities and they are performing the behavior. Such role models explained to other caregivers how they manage practicing the behavior in their homes, addressing factor others’ behavior (BCT 9, “inform about others’ behavior”) targeting factor behavior of others.

The fourth element of handwashing with soap behavior involved provision of cues to action (i.e. bracelets and bibs with handwashing message) to act as a reminder to caregivers about practicing the behavior in all the four critical times of handwashing targeting factor remembering (“use of memory aids and environmental prompts”, BCT 34).

The fifth activity involved encouraging the caregivers to make public pledge amongst themselves and in front of other community members addressing factors others’ behavior and prompt public commitment. Making public commitment to wash hands with soap demonstrated that others are already performing the behavior (“inform about others’ behavior”, BCT 9). In addition, the caregivers made the commitment by placing a plate with their hand print on a noticeable place within their household to show their friends/visitors about their commitment to the behavior (“making public commitment”, BCT 10) which also acted as a reminder to the

caregivers to practice the behavior ("use of memory aids and environmental prompts", BCT 34). Furthermore, to appreciate their adherence to the behavior, caregivers were given certificate at the last cluster meeting about handwashing which they put at an open place within their household, an indication of their commitment to the behavior. Sixth activity: Caregivers who sustained the behavior were rewarded addressing behavior factor costs and benefits ("use of subsequent reward," BCT 6).

Washing of kitchen utensils with soap

The first activity had a poster and a puzzle game exercise where caregivers were asked to put the cards with different images in the recommended order that is followed when preparing complementary food (i.e. porridge). The game and the poster addressed factors health knowledge through a discussion that highlighted the importance of washing utensils with soap ("present facts", BCT 1).

The second activity targeted confidence in performance and others' behavior factors through practical demonstration on the effectiveness of using soap in removing germs from utensils ("demonstrate and model behavior", BCT 17). Role models identified during household visits discussed and encouraged others on how they managed to practice the behavior ("inform about others' behavior", BCT 9 and "prompt to talk to others", BCT 7).

The third activity was about encouraging the caregivers to make public commitment related to washing utensils with soap addressing factors others' behavior and prompt public commitment. Making public commitment to wash utensils with soap showed that others are already performing the behavior ("inform about others' behavior", BCT 9). In addition, the caregivers made the commitment by placing a banting that contained an image about washing utensils with soap plate that was placed on a wall within the household to show their

friends/visitors about their commitment to the behavior ("making public commitment", BCT 10). The banting also acted as a reminder to the caregivers to practice the behavior ("use of memory aids and environmental prompts", BCT 34).

Keeping kitchen utensils on an elevated place

The first activity was a discussion among the caregivers about two types of poster illustrations i.e. 1. Showing good storage of utensils inside and outside the house (i.e. utensils on a raised place); 2. Showing poor storage of utensils inside and outside the house (i.e. utensils on the floor/ground). The illustrations sparked a debate about hygienic storage of the utensils. It encouraged the caregivers to talk to one another and provided knowledge ("present facts", BCT 1 and "prompt to talk to others", BCT 7). Thus it addressed the behavioral factor health knowledge. The second activity was a practical demonstration about dish rack construction to raise the confidence in performance ("provide infrastructure", BCT 16). The community volunteers guided the caregivers on how they can construct a dish rack with local resources and this was reinforced during household visits ("prompt behavioral practice," BCT 19). Those who already had dish racks encouraged others on the benefits and how they managed the behavior ("inform about others' behavior", BCT 9). In addition, they discussed with their colleagues on how they dealt with relapses ("prompt coping with relapse," BCT 25). Third activity: this activity addressed the remembering factor where the caregivers placed a banting on the wall with an image about keeping utensils on an elevated place ("use of memory aids and environmental prompts", BCT 34). Fourth activity: Caregivers made a public pledge by signing a pledge card, committing themselves to always practice the targeted food hygiene behaviors ("making public commitment", BCT 10). Fifth activity: Caregivers who sustained the behavior were rewarded addressing behavior factor costs and benefits ("use of subsequent reward," BCT 6).

Reheating of left-over food

The first activity was about a group discussion emphasizing on the positive feelings associated with reheating of left-over food ("Describe feelings about performing and about consequences of the behavior", BCT 8). Those who were already reheating their left over food encouraged others on the benefits and how they managed to perform the behavior ("inform about others' behavior", BCT 9). In addition, the role models discuss what propels them to always feed their children reheated left over food and this included consequences of not performing the behavior ("Prompt anticipated regret", BCT 12). With the use of poster illustrations, caregivers were encouraged on how they can deal with challenges (e.g. inadequate firewood) that hinders them from reheating their left over food ("confidence in performance" BCTs 18 and 22).

Feeding of children by the caregivers

With the use of role models, caregivers encouraged one another at cluster meetings that others are already performing the behavior ("inform about others' behavior", BCT 9). In addition, caregivers made commitment in. public to continue performing the behavior ("prompt public commitment," BCT 10). In cluster meetings, caregivers practically demonstrated to one another on how they can achieve the behavior which included concepts on how to deal with relapses ("Confidence in performance," BCTs 18 and 25).
